# Energy-Selective X-Ray Detection Using Chemically Tunable High-Z Nanocomposites

**DOI:** 10.3390/ma18174118

**Published:** 2025-09-02

**Authors:** Inga Pudza, Kaspars Pudzs, Andrejs Tokmakovs, Aleksandr Kalinko, Alexei Kuzmin

**Affiliations:** 1 Institute of Solid State Physics, University of Latvia, Kengaraga Street 8, LV-1063 Riga, Latvia; kaspars.pudzs@cfi.lu.lv (K.P.); andrejs.tokmakovs@cfi.lu.lv (A.T.); aleksandr.kalinko@desy.de (A.K.); 2 Deutsches Elektronen-Synchrotron (DESY), A Research Centre of the Helmholtz Association, Notkestrasse 85, D-22607 Hamburg, Germany

**Keywords:** SrWO_4_, CdWO_4_, tungstates, hybrid organic–inorganic X-ray detectors, X-ray sensing, X-ray imaging

## Abstract

Hybrid organic–inorganic materials incorporating high-*Z* nanocompounds represent an emerging area of research with high, cost-effective potential for radiation detection applications, owing to their ability to enable unprecedented architectures and functional devices. Herein, we introduce a new hybrid system composed of tungstate nanoparticles (SrWO_4_ or CdWO_4_) blended with P3HT:PCBM, engineered for direct X-ray detection without the need for external bias. The nanocrystalline tungstates were synthesized through a hydrothermal route. X-ray diffraction and scanning electron microscopy were employed to characterize the nanoparticle structure and morphology, respectively. Incorporation of high-*Z* tungstate nanoparticles was found to substantially enhance detector sensitivity within specific energy ranges, with performance tunable by varying the tungstate composition. The use of the fabricated detectors was demonstrated for both spectroscopic and imaging applications.

## 1. Introduction

Nanomaterial-based X-ray detectors constitute a rapidly developing area of research, as they enable high spatial resolution and can be implemented using various compounds at reduced cost [1,2,3,4,5,6]. Depending on the properties of the nanomaterial, either direct detectors (based on X-ray induced current) or indirect detectors (based on X-ray excited optical luminescence) can be realized [2,7]. Hybrid organic–inorganic detectors form a distinct class of materials with versatile capabilities for X-ray detection, which has drawn significant attention over the past decade [3,8,9]. These detectors offer several advantages, such as the combination of organic semiconductors, which feature low production cost, ease of fabrication, flexible shape, and low operating voltage (<10 V), with (nano-)compounds containing high-*Z* elements [2,10,11,12]. Incorporating high-*Z* elements increases the X-ray cross-section and affects the detector’s spectral selectivity, thereby improving its absorption efficiency and sensitivity while maintaining the advantageous physical properties of the organic host matrix [3,13]. Various inorganic materials, including metallic Ta [14], Bi_2_O_3_ [3,14,15,16], PbS [17], Gd_2_O_2_S:Tb [8], CsI(Tl) [18], have been proposed for use in hybrid organic–inorganic systems for X-ray detection. An emerging class of materials in this context is metal halide perovskites [19]. However, the search for optimal systems suitable for widespread application, ranging from medical diagnostics and security scanners to industrial inspection and state-of-the-art scientific research using synchrotrons and free electron lasers, remains a challenge in the field [9,20,21,22,23].

Among various inorganic compounds, tungstates with the general formula AWO_4_ [24] (A is a divalent ion such as Sr, Ba, Pb, Mn, Co, Ni, or Cd) have yet to be explored for use in hybrid organic–inorganic X-ray detectors, despite the high atomic number of tungsten (*Z* = 74). At the same time, CaWO_4_, CdWO_4_, and PbWO_4_ are well-known as X-ray phosphors and scintillator materials [2,25]. Tungstates, including their solid solutions and multicomponent derivatives [26], offer a wide range of opportunities for optimizing hybrid detector properties through variation in chemical composition and crystallinity. The ability to vary the *Z*-number of the A-site metal ion over a wide range or even replace it with rare-earth elements [27,28] offers a straightforward and convenient way to tune both the absorption efficiency in specific X-ray energy ranges and the optical properties of the tungstate under X-ray illumination, as required for direct detection. Recently, we explored two nanotungstates (CaWO_4_ and ZnWO_4_) for application in organic–inorganic X-ray detectors. These materials showed promising, though relatively slow, responses to X-ray radiation [29], resulting in a significant loss of energy resolution in spectroscopic applications (see Figure 5 in [29]). Therefore, the search for improved materials should continue.

In this study, two nanocrystalline tungstates, SrWO_4_ and CdWO_4_, were synthesized as potential X-ray absorbers for hybrid organic–inorganic direct X-ray detectors. These compounds, exhibiting scheelite and wolframite crystallographic structures, respectively [24], were characterized using X-ray diffraction (XRD) and scanning electron microscopy (SEM). X-ray detectors operating without an external bias voltage were fabricated using a mixture of nanotungstate powders and a P3HT:PCBM blend, demonstrating improved responses compared to those reported in [29]. Their application for X-ray detection in both spectroscopic and imaging modes was evaluated using tunable synchrotron radiation. The differences in spectral response between detectors based on SrWO_4_ and CdWO_4_ nanoparticles were analyzed across various X-ray energies.

## 2. Materials and Methods

### 2.1. Synthesis and Characterization of Tungstate Nanoparticles

SrWO_4_ and CdWO_4_ nanoparticles (NPs) were synthesized via a hydrothermal method [30]. Citric acid (C_6_H_8_O_7_) was used as a capping agent to control grain growth [31]. All chemicals were of analytical grade and used as received without further purification.

Initially, 3 mmol of Sr(NO_3_)_2_ (99.97%, Alfa Aesar, Haverhill, MA, USA) or CdCl_2_ (99.99%, Sigma-Aldrich, St. Louis, MO, USA) and Na_2_WO_4_·2H_2_O (≥99%, Alfa Aesar) were separately dissolved in deionized water. Then, citric acid (1.5 mmol) was added to the solution of strontium (or cadmium) salt. The resulting mixed solution was subsequently added to the Na_2_WO_4_·2H_2_O solution. The pH of the final mixture was adjusted to 9 (or 8 for the cadmium-containing solution) using an aqueous NaOH (≥98%, Sigma-Aldrich) solution. The mixture was stirred magnetically for 30 min. A portion of the resulting solution (16 ml) was transferred into a 25 mL Teflon-lined stainless steel autoclave and subjected to hydrothermal treatment at ∼160 °C for 24 h, with a subsequent natural cooling to room temperature (RT) (SrWO_4_-3 & CdWO_4_-3). The remaining solution was left at RT for 24 h to prepare the reference samples (SrWO_4_-2 and CdWO_4_-2). For comparison, an additional synthesis was conducted without citric acid and without hydrothermal treatment, using a simple co-precipitation method at RT (SrWO_4_-1 and CdWO_4_-1). The resulting tungstate precipitates were washed with distilled water. The samples obtained from hydrothermal treatment were repeatedly washed and centrifuged using the following sequence: distilled water, isopropanol, acetone, and chlorobenzene, and were finally dried in air at 75 °C.

X-ray powder diffraction (XRD) was used to characterize the phase composition and crystallinity of all samples. The measurements were performed using a benchtop Rigaku MiniFlex 600 powder diffractometer (Rigaku, Tokyo, Japan) with Bragg–Brentano geometry. X-rays were produced by a tube with copper anode (K*_α_* radiation), operated at 40 kV and 15 mA. Crystallite sizes were estimated via Rietveld refinement using the Profex software (version 5.0) [32].

Scanning electron microscopy (SEM) was employed to examine the sample morphology. A Helios 5 UX microscope (Thermo Fisher Scientific, Waltham, MA, USA), equipped with Elstar in-lens TLD-SE detector (Thermo Fisher Scientific, Waltham, MA, USA), was used in immersion mode at 2.0 kV.

### 2.2. Fabrication of Hybrid Organic–Inorganic X-Ray Detectors

ITO (In_2_O_3_:Sn)-coated glass substrates with a size of 25 × 25 mm^2^ and a sheet resistance of 5 Ω/□ (Präzisions Glas & Optik GmbH, Iserlohn, Germany) were used as substrates to fabricate the X-ray detectors. A 40 nm thick layer of poly(3,4-ethylenedioxythiophene)-poly(styrenesulfonate) (PEDOT:PSS; Heraeus Al4083, Leverkusen, Germany) was employed as the hole transport and electron blocking layer. This layer was spin-coated in air at 2500 rpm for 40 s with an acceleration of 2500 rpm/s, followed by annealing at 150 °C for 10 min.

Next, tungstate powder (SrWO_4_-3 and CdWO_4_-3) was mixed with a P3HT:PCBM solution (1:1 weight ratio) in anhydrous chlorobenzene (99.8%, Sigma-Aldrich) and sonicated for 1 h, yielding a suspension of tungstate NPs and P3HT:PCBM. Various NPs:P3HT:PCBM weight ratios were then investigated to optimize X-ray attenuation while maintaining uniform film morphology. The weight ratio used to produce the final SrWO_4_:P3HT:PCBM solution was 1:1:1, whereas it was adjusted to 2:1:1 for CdWO_4_:P3HT:PCBM. Thin films were deposited by blade-casting the suspension onto substrates preheated to 75 °C, followed by annealing at 140 °C for 15 min to crystallize the P3HT:PCBM blend.

A 5 nm-thick hole-blocking layer of 4,7-diphenyl-1,10-phenanthroline (BPhen, Sigma-Aldrich 133159), followed by a 100 nm-thick aluminum (Al) electrode, was deposited on top of the hybrid layer by thermal evaporation. Deposition was carried out under vacuum at a pressure below 7 × 10^−6^ mbar. The aluminum electrodes were patterned to form six independent “active pixels” with dimensions of 4 × 4 mm^2^, enabling individual testing. The resulting hybrid organic–inorganic X-ray detector exhibited a five-layer structure: ITO/PEDOT:PSS/NPs:P3HT:PCBM/BPhen/Al. To enhance environmental stability, the detectors were encapsulated with a glass cover.

### 2.3. Testing of X-Ray Detectors for Different Applications

**Figure 1:**
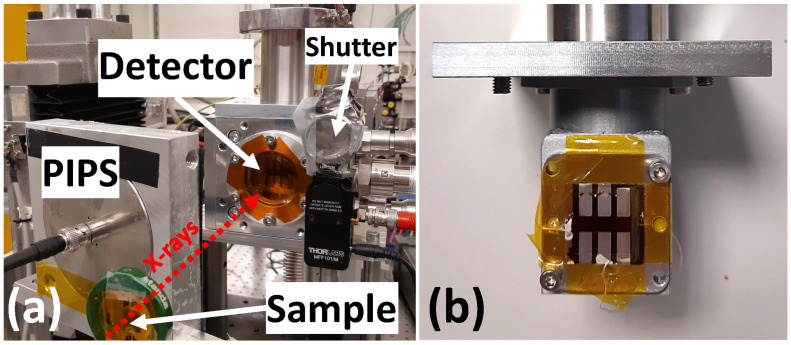
(**a**) Experimental setup at the PETRA-III P64 beamline. The hybrid detector, shutter, sample, and PIPS diode are visible. The direction of the incoming X-rays is indicated. (**b**) Enlarged view of the hybrid detector. Six independent pixels with aluminum contacts are visible.

Testing of the X-ray detectors was carried out at the DESY P64 Advanced X-ray Absorption Spectroscopy beamline (Hamburg, Germany) [33]. The PETRA-III storage ring operated in top-up mode with 40 bunches at an energy of *E* = 6 GeV and a ring current of *I* = 100 mA. The desired photon energy from the undulator source was selected using a double-crystal monochromator with Si(111) crystals. The X-ray beam incident on the sample had a size of 1 × 1 mm^2^. The intensity $I_{0}$ of the incoming monochromatic X-rays was first monitored with an ionization chamber and subsequently measured using the hybrid organic–inorganic direct X-ray detector. The detector was placed in a vacuum chamber and connected to a Keithley 428 current amplifier (Keithley Instruments, Cleveland, Ohio, USA). A shutter positioned in front of the detector enabled X-ray on/off measurements. To prevent potential photoelectric effects from ambient light in the experimental hutch, all experiments were conducted in the dark. The experiment setup is shown in Figure 1.

The detector signal, $I_{detector}$, was recorded using the ionization chamber monitoring system of the P64 beamline. The measured photocurrent values ranged from 0.2 to 2.0 nA. When required, a metal foil sample (Ni or Mo) was additionally placed in front of the detector. Simultaneously, X-ray fluorescence from the sample was detected using a Passivated Implanted Planar Silicon (PIPS) detector (Canberra, Meriden, CT, USA).

A motorized two-axis linear translation stage was used in the imaging experiments to enable precise positioning (±10 µm), allowing raster scans of two samples composed of overlapping metal foils (Ni+Cu and Nb+Mo) to demonstrate the elemental sensitivity of the hybrid detector. The sample scanning was performed in a direction orthogonal to the X-ray beam. Each image comprised 25 × 25 points, with a step size of 1 mm in both directions, resulting in a total image area of 25 × 25 mm^2^. The acquisition time was 1 s/point. The foil thicknesses were 4 µm for Ni, 5 µm for Cu, 20 µm for Nb, and 15 µm for Mo. These thicknesses were intentionally selected to achieve an absorption edge jump of about 1 in X-ray absorption experiments. This allowed us to maximize the signal measured in transmission mode during imaging and minimize distortions in X-ray absorption fine structure during spectroscopic measurements. In the imaging experiments, the PIPS detector was positioned in a backscattering geometry (Figure 1a) to detect X-rays scattered by the hybrid detector.

## 3. Results and Discussion

### 3.1. Characterization of Tungstate Nanoparticles

**Figure 2:**
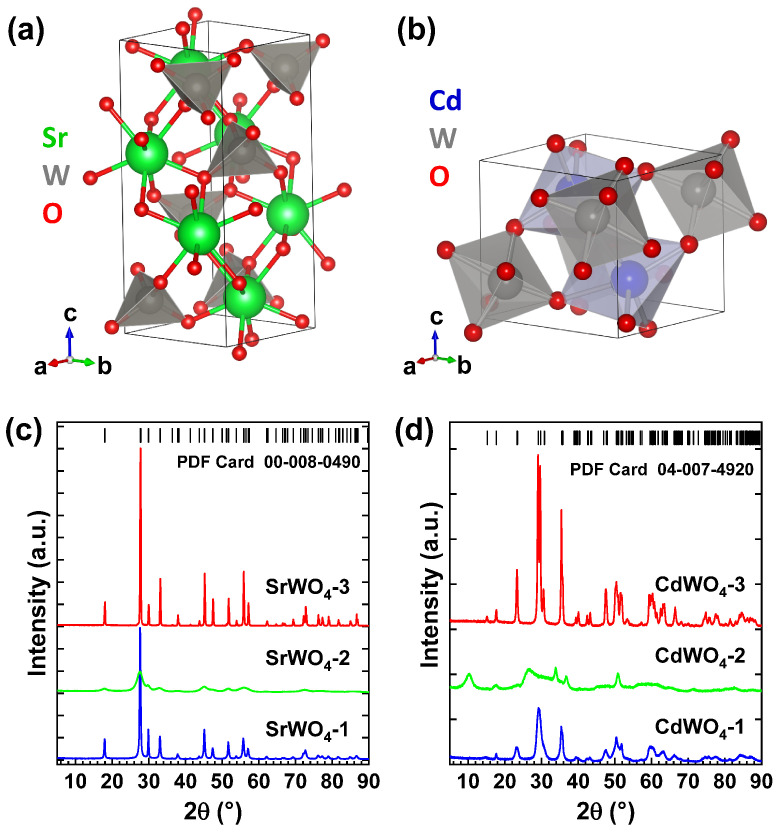
Crystallographic structure of scheelite SrWO_4_ (**a**) and wolframite CdWO_4_ (**b**). X-ray diffraction patterns of SrWO_4_ (**c**) and CdWO_4_ (**d**) nanoparticles after co-precipitation at RT (SrWO_4_-1 and CdWO_4_-1), synthesized with citric acid and left at RT (SrWO_4_-2 and CdWO_4_-2), synthesized with citric acid and heated in the autoclave for 24 h at 160 °C (SrWO_4_-3 and CdWO_4_-3). Peak positions corresponding to the PDF cards for tetragonal SrWO_4_ (PDF Card 00-008-0490) and monoclinic CdWO_4_ (PDF Card 04-007-4920) are shown at the top for comparison.

The crystallographic structures of bulk tungstates SrWO_4_ and CdWO_4_ are shown in Figure 2a,b. SrWO_4_ adopts a tetragonal scheelite-type structure with the space group $I4_{1}/a$. It consists of WO_4_ tetrahedra with strontium ions located between them and coordinated with eight oxygen ions [34]. In contrast, CdWO_4_ crystallizes in a monoclinic wolframite-type structure with the space group P2/c, built from distorted WO_6_ and CdO_6_ octahedra linked by shared edges to form infinite zigzag chains along the *c*-axis [35]. Both tungstates can also be synthesized in the nanocrystalline form [30,36,37].

XRD patterns of three different SrWO_4_ and CdWO_4_ nanocrystalline samples are shown in Figure 2c,d. They were synthesized without (SrWO_4_-1 and CdWO_4_-1) and with citric acid at room temperature (RT) (SrWO_4_-2 and CdWO_4_-2), as well as using hydrothermal treatment at ∼160 °C for 24 h in the autoclave (SrWO_4_-3 and CdWO_4_-3). The nanoparticles of both tungstates prepared with citric acid at RT (SrWO_4_-2 and CdWO_4_-2) exhibit weak crystallinity, with an average size of about 6 nm for the SrWO_4_-2 sample. The size of CdWO_4_ nanoparticles was not determined due to a strong peak broadening and the presence of XRD peaks from impurities, e.g., trisodium citrate hydrates [38]. The XRD patterns of nanoparticles synthesized without citric acid and left at RT, or synthesized with citric acid and subjected to hydrothermal treatment, display numerous Bragg peaks that can be attributed to the pure tungstate phases (PDF Card 00-008-0490 for SrWO_4_ and PDF Card 04-007-4920 for CdWO_4_). The hydrothermally grown nanotungstates had the average crystallite sizes of ∼97 nm for the SrWO_4_-3 sample and ∼36 nm for the CdWO_4_-3 sample, as determined using the Rietveld refinement. In comparison, the sizes of nanoparticles synthesized without citric acid at RT were ∼30 nm for the SrWO_4_-1 sample and 10 nm for the CdWO_4_-1 sample.

All XRD reflections for hydrothermally treated samples can be indexed to tetragonal SrWO_4_ phase (space group $I4_{1}/a$) with lattice constants *a* = *b* = 5.420(1) Å and *c* = 11.958(1) Å and to monoclinic CdWO_4_ phase (space group P2/c) with lattice parameters *a* = 5.030(1) Å, *b* = 5.866(1) Å, *c* = 5.079(1) Å, and β = 91.510(2)°.

**Figure 3:**
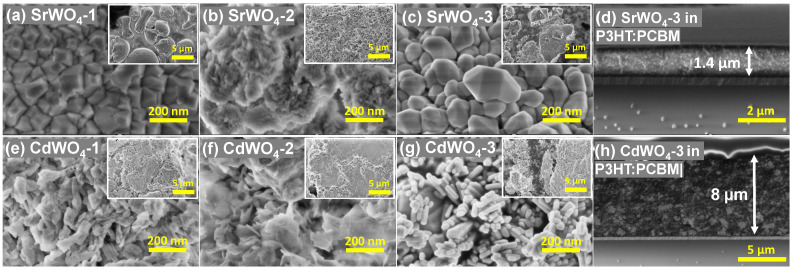
SEM micrographs of SrWO_4_ and CdWO_4_ nanoparticles after co-precipitation at RT (**a**,**e**), synthesized with citric acid and left at RT (**b**,**f**), synthesized with citric acid and heated in the autoclave for 24 h at 160 °C (**c**,**d**,**g**,**h**). Cross-sectional SEM images of the fabricated hybrid detectors, showing tungstate NPs embedded in the P3HT:PCBM matrix, are also presented in (**d**,**h**). The scale bars are 200 nm in (**a**–**c**,**e**–**g**), 5 µm in the insets in (**a**–**c**,**e**–**g**), 2 µm in (**d**), and 5 µm in (**h**).

Figure 3 shows SEM micrographs of the nanotungstates at different magnifications, illustrating their morphology. Products synthesized at RT with citric acid form fine powders consisting of agglomerated particles (Figure 3b,f). Meanwhile, nanocrystallites begin to form at RT in the absence of citric acid (Figure 3a,e), although their shapes are not well defined, especially in the case of the CdWO_4_-1 sample.

Hydrothermal treatment at ∼160 °C markedly enhances the crystallinity, as indicated by the well-defined facets of the tungstate crystallites shown in Figure 3c,g. Nevertheless, the shape of crystallites is different due to the different crystallographic structures of the two tungstates [24]. The SEM images of the sample cross-section are shown in Figure 3d,h. Here, the glass substrate is visible at the bottom of both images. The thickness of SrWO_4_-3 in P3HT:PCBM blend is about 1.4 µm, whereas that of CdWO_4_-3 is about 8 µm. In both cases, a rather homogeneous distribution of tungstate nanoparticles in the organic matrix is observed.

### 3.2. Hybrid Organic–Inorganic X-Ray Detector Response

**Figure 4:**
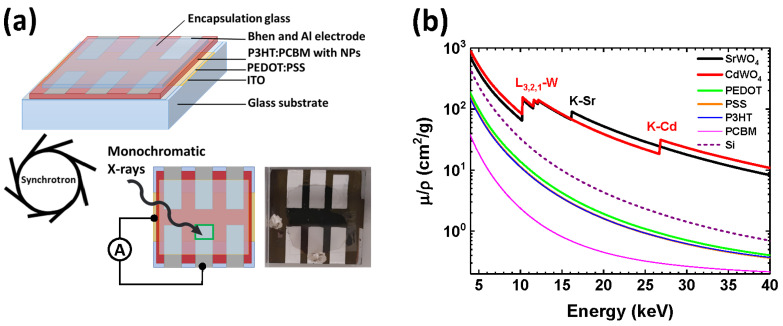
Schematic illustration of the fabricated hybrid organic–inorganic direct-conversion X-ray detector and its wiring for bias-free operation from [29] (**a**). The active pixel of the detector is indicated by a green rectangle. X-ray mass attenuation coefficients of SrWO_4_, CdWO_4_, PEDOT, PSS, P3HT, PCBM, and Si as a function of photon energy (**b**).

Bulk heterojunction organic solar cells frequently employ the P3HT:PCBM blend as the active layer [39], owing to its good efficiency in the visible and UV regions. However, organic polymers are poor X-ray absorbers, particularly in the hard X-ray range, due to their low attenuation coefficients. Incorporating inorganic nanoparticles with high atomic numbers (*Z*) provides a strategy to overcome this limitation [3]. Accordingly, we developed hybrid organic–inorganic X-ray detectors consisting of five layers, ITO/PEDOT:PSS/NPs:P3HT:PCBM/BPhen/Al (NPs = SrWO_4_ or CdWO_4_), deposited on glass substrates and protected by a glass encapsulation (Figure 4a). A reference device without nanoparticles [29] was also investigated for comparison.

Each detector contains six independent pixels, with only one pixel used in each experiment. X-ray mass attenuation coefficients for SrWO_4_, CdWO_4_, PEDOT, PSS, P3HT, PCBM, and silicon were calculated using tabulated data from Ref. [40]. Their energy dependence is compared in Figure 4b. The benefit of stronger X-ray absorption due to the presence of high-*Z* elements is evident above 10 keV. It is also worth noting that our hybrid P3HT:PCBM-based detectors, like polymer solar cells [41], are able to operate in a self-powered mode, requiring no external bias voltage.

X-ray absorption spectra of elements exhibit sharp rises, known as absorption edges, which occur at specific X-ray energies corresponding to the binding energy of core-level electrons. For example, strontium (*Z* = 38) and cadmium (*Z* = 48) have K-edges at 16,105 eV and 26,711 eV, respectively, while the heavier element tungsten (*Z* = 74) exhibits a series of three L_1,2,3_-edges at 10,207 eV, 11,544 eV, and 12,100 eV [42]. A sharp increase in absorption just beyond these edges can be exploited to optimize the spectral sensitivity of detectors within a desired energy range. Additionally, a pronounced absorption resonance known as the “white line” (WL) is often observed immediately above the absorption edge in X-ray absorption spectra of some compounds. This feature is common in the X-ray absorption spectra of tungsten and rare-earth compounds at the metal L_2,3_-edges and arises from the quasi-localized nature and high density of unoccupied 5d metal states [43,44,45]. In this study, we utilized the WL at the W L_3_-edge (located at 10,212 eV) to enhance the signal in detectors incorporating tungstates.

Monochromatic synchrotron radiation at three photon energies (9500 eV, 10,212 eV, and 20,000 eV) was used to irradiate the fabricated hybrid detectors, enabling evaluation of the influence of tungstate NPs in the active layer on detector behavior. The first energy (9500 eV) lies below the W L_3_-edge, the second (10,212 eV) corresponds to the WL maximum, and the third (20,000 eV) falls between the K-edges of Sr and Cd.

X-ray photon absorption in matter leads to the formation of electron–hole pairs through the internal photoelectric effect, accompanied by avalanche-like secondary electron generation. In the detector, these carriers drift toward the electrodes and are registered as an electrical signal. In hybrid systems, the charge transport and recombination processes are influenced by the interface quality and the compatibility between the organic and inorganic components. Nanoparticles with dimensions under 100 nm can promote better active layer homogeneity and facilitate more effective charge extraction.

**Figure 5:**
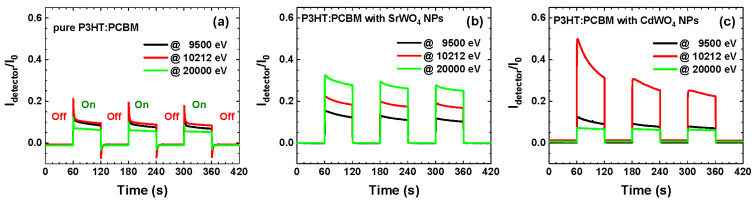
X-ray response ($I_{detector}$/I_0_) of pure P3HT:PCBM (adapted from [29]) (**a**), SrWO_4_+P3HT:PCBM (**b**), and CdWO_4_+P3HT:PCBM (**c**) hybrid detectors under successive X-ray on/off exposures.

Time-dependent X-ray experiments (Figure 5) were performed using two hybrid detectors under repeated X-ray exposure cycles (on/off) with a 120 s period and compared to the response of a pure organic P3HT:PCBM detector, adapted from [29]. The normalized X-ray response was calculated as the intensity ratio $I_{detector}\left(E\right)/I_{0}\left(E\right)$, where $I_{0}\left(E\right)$ corresponds to the incident X-ray intensity measured by the ionization chamber, and $I_{detector}\left(E\right)$ is the signal detected by the respective detector. All three detectors exhibit good sensitivity to X-rays, producing responses close to a “square-shaped” signal. It is worth noting that a different response shape, resembling a “saw-tooth” profile, has been observed in some purely organic [12] and hybrid [46] X-ray detectors. Since the absolute values of the photocurrent are influenced by the fabrication quality of the detectors, we focus on comparing their relative responses at three selected X-ray energies (9500 eV, 10,212 eV, and 20,000 eV).

The X-ray response of a pure organic P3HT:PCBM detector is shown in Figure 5a. Because organic polymers consist of light elements, their X-ray attenuation coefficient decreases rapidly as photon energy increases (Figure 4b), leading to diminished sensitivity at high energies. Consequently, the photocurrent is nearly identical at 9500 eV and 10,212 eV but drops significantly at 20,000 eV. Upon irradiation, the current rises sharply to a maximum and then relaxes, decreasing by roughly 30–50% within the first few seconds. Once the X-ray beam is shuttered, the dark current exhibits a brief negative overshoot before stabilizing at its baseline. The abrupt photocurrent changes observed at shutter switching points are most likely associated with charge trapping/detrapping at the material–electrode interfaces and with space-charge phenomena [3,6,47].

Figure 5b,c shows that the normalized responses of hybrid detectors with SrWO_4_ and CdWO_4_ nanoparticles converge to similar values after stabilization and align well with results from other hybrid devices [48,49]. Importantly, unlike pure organic detectors, these hybrids display stable photocurrent traces without sharp variations at X-ray shutter switching. The enhancement in response at 10,212 eV originates from the strong absorption resonance (”white line”) just above the W L_3_-edge [44,45], with additional absorption contributions from the tungsten L_1,2,3_-edges at 10,207 eV, 11,544 eV, and 12,100 eV (Figure 4b). In the SrWO_4_-based detector (Figure 5b), absorption at the Sr K-edge (16,105 eV) further boosts the high-energy response, giving a photocurrent at 20,000 eV nearly twice that at 9500 eV. By contrast, since the Cd K-edge is located much higher in energy at 26,711 eV (Figure 4b), the CdWO_4_-based detector exhibits a reduced response at 20,000 eV (Figure 5c).

### 3.3. The Use of X-Ray Detectors for Spectroscopic and Imaging Applications

**Figure 6:**
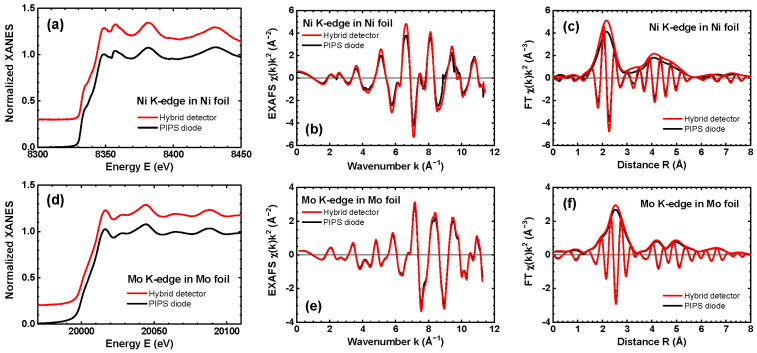
Comparison of the Ni (**a**–**c**) and Mo (**d**–**f**) K-edge XANES μ(E) and EXAFS spectra $\chi \left(k\right)k^{2}$ as well as Fourier transforms of EXAFS spectra for nickel and molybdenum foils measured using the hybrid SrWO_4_-P3HT:PCBM and PIPS detectors.

An example of the use of the hybrid SrWO_4_-P3HT:PCBM detector for spectroscopic applications is presented in Figure 6. X-ray absorption spectra of nickel and molybdenum metal foils, measured at the Ni and Mo K-edges, respectively, were simultaneously recorded using the hybrid detector (in transmission mode) and a PIPS diode (in fluorescence mode, for comparison). This setup demonstrates the detector sensitivity across two distinct energy ranges—around 8 keV and 20 keV. The XAS spectra were acquired in continuous scan mode over an energy range of 600 eV (2000 points per spectrum), with an exposure time of about 0.1 s/point.

Figure 6a,d presents the normalized X-ray absorption near edge structure (XANES) spectra μ(E), which display well-resolved and comparable oscillations and shoulders for both detectors. Notably, significant broadening of the XANES oscillations was observed in our previous work (see Figure 5 in [29]) for a detector based on ZnWO_4_ nanoparticles. This broadening was attributed to the slower temporal response of ZnWO_4_ compared to the other tungstates tested (see the shape of the “on-off” signal in Figure 5 and compare it with Figure 4 in [29]).

Figure 6b,e compares the extracted extended X-ray absorption fine structure (EXAFS) spectra $\chi \left(k\right)k^{2}$ at the Ni and Mo K-edges. The wavenumber *k* is given by $k=\sqrt{\left(2m_{e}/\hbar^{2}\right)\left(E-E_{0}\right)}$, where $m_{e}$ denotes the electron mass, *ℏ* is the reduced Planck constant, and $E_{0}$ is the threshold energy, corresponding to the kinetic energy of a free electron at zero momentum. The slightly lower amplitude of the EXAFS oscillations detected by the PIPS detector is due to the so-called “self-absorption” effect, which occurs in thick samples as foils measured in fluorescence detection mode [50]. Nevertheless, good agreement is observed between the EXAFS spectra measured at both absorption edges by the two detectors at high wavenumbers *k*, where the signal is multiplied by the $k^{2}$ weighting factor. This result demonstrates the large dynamic range of the hybrid detector. Additionally, good agreement between the Fourier transforms of the EXAFS spectra (Figure 6c,f) is observed up to 8 Å, indicating good sensitivity to smaller high-frequency EXAFS oscillations originating from the outer coordination shells of metal atoms in both foils. A more detailed comparison of the EXAFS spectra shows slightly better agreement for the Mo K-edge than for the Ni K-edge. This difference can be attributed to the higher sensitivity of the hybrid detector at greater photon energies, where absorption by both Sr and W atoms contributes to X-ray attenuation (Figure 4b). Indeed, the Ni K-edge energy (8333 eV) lies below the W L_3_-edge (10,204 eV), while the Mo K-edge energy (20,000 eV) lies above the W L_1,2,3_ edges (10,204 eV, 11,544 eV, and 12,100 eV) and the Sr K-edge (16,105 eV).

**Figure 7:**
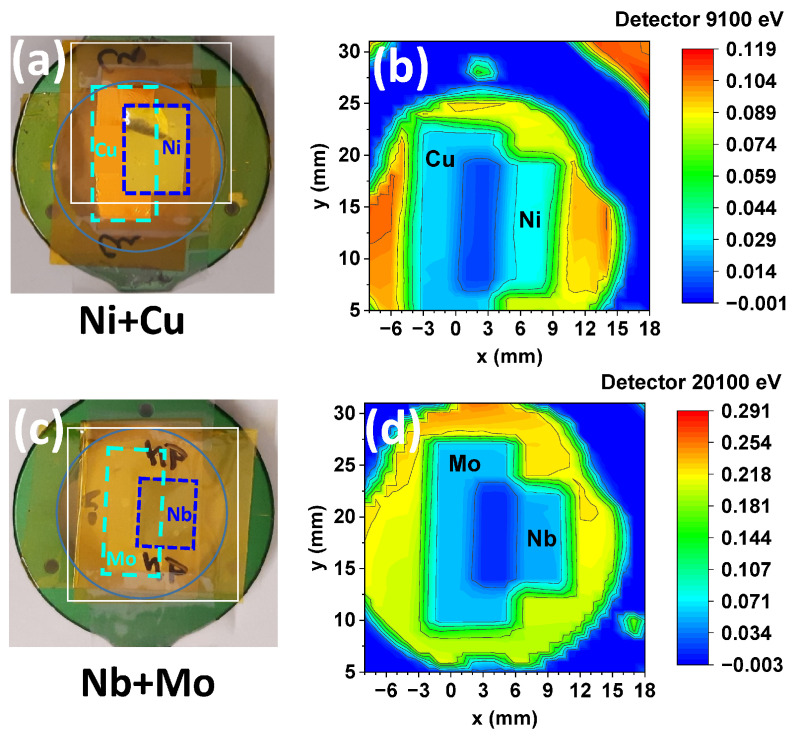
Imaging of two test samples, each consisting of a pair of overlapping metal foils ((**a**) Ni+Cu and (**c**) Nb+Mo), using the hybrid SrWO_4_-P3HT:PCBM detector. Spectral image (**b**) was recorded at an energy of 9100 eV (above the Ni and Cu K-edges), and spectral image (**d**) was recorded at an energy of 20,100 eV (above the Nb and Mo K-edges).

Imaging experiments were carried out using two test samples made from two pairs of rectangular metal foils (Ni+Cu and Mo+Nb). These pairs were selected to evaluate the ability to resolve neighboring elements in the Periodic Table. The foils were attached to a round metallic sample holder using Kapton film (Figure 7a,c). Images measured with the SrWO_4_-P3HT:PCBM hybrid at 9100 eV (i.e., above the K-edges of Ni and Cu) and at 20,100 eV (i.e., above the K-edges of Nb and Mo) are shown in Figure 7b,d. In these images, the blue color corresponds to the lowest X-ray intensity, and the red color to the highest. As seen, the lowest intensity (dark blue color) appears behind the sample holder and in the central region of the image, where the two metal foils overlap and thus increase the overall X-ray absorption. In regions where the two foils do not overlap, they appear light blue, and their outlines are clearly visible. The highest intensities are observed outside the sample holder in Figure 7b and in areas containing only the Kapton film. The higher absolute signal observed at 20,100 eV is attributed to increased absorption due to the presence of strontium atoms in the detector, whose K-edge is located at 16,105 eV.

**Figure 8:**
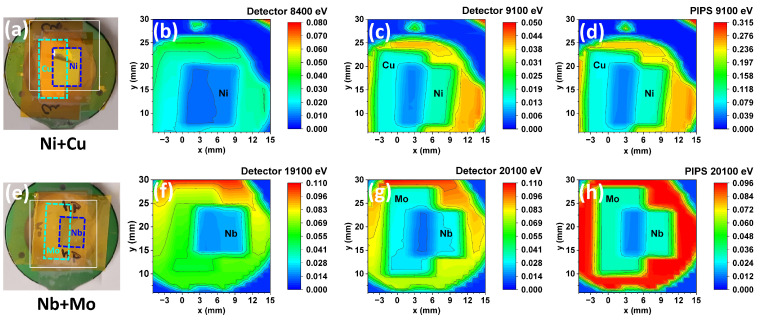
Imaging of two test samples, each consisting of a pair of overlapping metal foils ((**a**) Ni+Cu and (**e**) Nb+Mo), using the hybrid CdWO_4_-P3HT:PCBM detector and a PIPS diode. Spectral images (**b**) and (**c**,**d**) of the Ni+Cu sample (**a**) were recorded at energies of 8400 eV (between the Ni and Cu K-edges) and 9100 eV (above the Cu K-edge), respectively. Spectral images (**f**) and (**g**,**h**) of the Nb+Mo sample (**e**) were recorded at energies of 19,100 eV (between the Nb and Mo K-edges) and 20,100 eV (above the Mo K-edge), respectively.

Images measured with the CdWO_4_-P3HT:PCBM hybrid detector at four different energies are presented in Figure 8b,c,f,g. For comparison, the images recorded simultaneously with the PIPS detector are also shown in Figure 8d,h. Note that PIPS was oriented towards the hybrid detector, not the sample, so its signal is proportional to the X-rays scattered by the detector. The first sample (Ni+Cu) was measured at 8400 eV, i.e., above the Ni K-edge but below the Cu K-edge, and at 9100 eV, i.e., above the K-edges of both Ni and Cu. As a result, the image at 8400 eV shows the strongest contrast at the position corresponding to the Ni foil (Figure 8b), while the image at 9100 eV displays the greatest contrast where the two foils overlap (Figure 8c). A good correlation is observed between the images measured at 9100 eV using the hybrid and PIPS detectors (Figure 8c,d).

Images of the second sample (Nb+Mo) were recorded at 19,100 eV, i.e., above the Nb K-edge but below the K-edge of Mo, and at 20,100 eV, i.e., above the K-edge of both Nb and Mo. The image at 19,100 eV (Figure 8f) reveals the Nb foil, while the image at 20,100 eV shows contrast for both foils (Figure 8g). Also, in this case, there is good agreement between the images obtained at 20,100 eV using the hybrid and PIPS detectors (Figure 8g,h). Note that the highest intensity in Figure 8h, detected by the PIPS detector from the Kapton film (red color), is due to the X-ray fluorescence from CdWO_4_ nanoparticles located inside the hybrid detector and excited at the W L-edges.

The results demonstrate the potential of hybrid organic–inorganic direct detectors for both spectroscopic and imaging applications. Furthermore, the ability to tailor the chemical composition of the inorganic nanoparticles enables optimization of X-ray absorption in specific energy ranges, thereby enhancing detector sensitivity. Tungstates of various compositions are promising candidates for this purpose, although the fabrication quality of the detectors still requires optimization.

## 4. Conclusions

An X-ray-sensitive hybrid organic–inorganic direct detector was fabricated using nanotungstate powders (SrWO_4_ or CdWO_4_) blended with P3HT:PCBM in a five-layer sandwich structure: ITO/PEDOT:PSS/NPs:P3HT:PCBM/BPhen/Al. The detector demonstrated bias-free operation for both spectral and imaging applications under monochromatic synchrotron radiation. Nanocrystalline SrWO_4_ and CdWO_4_ were synthesized via a hydrothermal method, both with and without citric acid as a capping agent, with the highest performance achieved for nanocrystallites prepared in the presence of citric acid.

The inclusion of tungstate nanoparticles containing high-*Z* elements significantly increased the sensitivity of the detector within specific energy ranges, determined by the chemical composition of the tungstate. Specifically, detectors based on strontium and cadmium tungstates exhibited enhanced responses due to increased X-ray absorption above the L_3,2,1_-edges of tungsten (10,207, 11,544, and 12,100 eV), as well as the K-edges of strontium (16,105 eV) and cadmium (26,711 eV). Therefore, hybrid detectors based on AWO_4_ tungstate nanoparticles, with variable chemical composition defined by the A-site cation, offer a cost-effective solution for X-ray applications.

## Data Availability

The original contributions presented in this study are included in the article. Further inquiries can be directed to the corresponding authors.

## Abbreviations

The following abbreviations are used in this manuscript:

EXAFS	extended X-ray absorption fine structure
ITO	indium tin oxide
NP	nanoparticle
P3HT:PCBM	Poly(3-hexylthiophene-2,5-diyl):Phenyl-C61-butyric acid methyl ester
PEDOT:PSS	Poly(3,4-ethylenedioxythiophene)-poly(styrenesulfonate)
PIPS	passivated implanted planar silicon
RT	room temperature
SEM	scanning electron microscopy
WL	white line
XANES	X-ray absorption near edge structure
XRD	X-ray diffraction
